# Perspectives on Mass Media and Governmental Measures during the 2020 COVID-19 Pandemic in a Romanian Sample of Healthcare Practitioners

**DOI:** 10.3390/healthcare10020191

**Published:** 2022-01-19

**Authors:** Daniela Reisz, Iulia Crișan

**Affiliations:** 1 Department of Neurology, University of Medicine and Pharmacy “Victor Babeș”, 300041 Timișoara, Romania; reisz.daniela@umft.ro; 2 Department of Psychology, West University of Timișoara, 300223 Timișoara, Romania

**Keywords:** COVID-19 infodemic, healthcare practitioners, mass media, governmental measures, vaccination priority, moral values

## Abstract

Objective: Along with the rapid spread of the COVID-19 pandemic around the globe, a proliferation of mass media information exposed the population to an infodemic with various implications documented worldwide. The present study analyzed Romanian healthcare practitioners’ (HCPs) appraisal of COVID-19 mass media information and governmental measures throughout 2020, ranking vaccination priorities and moral values. Methods: 97 HCP completed a cross-sectional survey with items referring to the COVID-19 pandemic in 2020. Results: Findings were consistent with other studies, indicating an overall negative appraisal of mass media information, which predicted anxiety and relaxation difficulties. Unlike other studies, our sample reported a moderate level of satisfaction with official measures in 2020, which was not related to their view on mass media information. The ranking of population categories in the vaccination order showed similarities with the governmental vaccination program in 2021. Despite placing freedom third after health and love in the hierarchy of values, HCPs showed a high tendency of limiting individual liberty for the common good. Conclusions: Results showed a dissociation between the overall negative appraisal of mass media information and the satisfaction with governmental measures in 2020. Romanian HCPs shared a secular perspective on moral values and assumed an authoritarian position.

## 1. Introduction

According to the World Health Organization, the COVID-19 pandemic has generated an infodemic (i.e., an abundance of digital or physical information that includes false or misleading news and causes confusion, disinformation, insecurity, and mistrust in government officials and health authorities) [1]. Besides mass media, the rapid spread of information through the Internet and social media contributed significantly to this phenomenon and hindered accurate and reliable information [2,3,4,5,6]. A meta-analysis on mainstream and social media information sources reflected the infodemic’s expansion, revealing 2311 reports from 87 countries containing COVID-19 related information, of which 82% were false [7]. Despite being a global phenomenon, research has shown that the infodemic manifested differently worldwide. International infodemiology studies [8,9] revealed a higher prevalence and a more rapid spread of bogus news, leading to a higher incidence of misinformation, in poorer countries. Romania, whose 2020 volume index of GDP per capita was about 30% below the average of EU countries [10], reaching 12,896 USD per capita, would therefore qualify as a poorer EU country. Among the implications of the infodemic, in Romania, we observed several phenomena that were documented worldwide: the proliferation of rumors about false treatment or prophylactic methods [6,11,12,13], the emergence of extremist discourse and conspiracy theories [5,14,15,16], and vaccine acceptance vs. hesitancy [17,18,19,20,21]. The need to empirically investigate such observations and the lack of COVID-19-related research on Romanian samples represented motivations for the present study.

Since mass administration of anti-COVID-19 vaccines is the most effective weapon against the pandemic, public vaccination acceptance is key to a successful immunization campaign. Emergent research has shown that, around the world, misinformation has negatively impacted vaccination adherence [9,22,23,24], being associated with higher levels of mistrust in official health policies [21,25,26]. Recent statistics also show that, while over 70% of the population in some European countries (e.g., Italy, The Netherlands) has received complete vaccination, in Romania, only 40.6% of the citizens were fully vaccinated at the beginning of 2022 [27]. The present study was motivated by the rather low incidence of vaccination in Romania and intended to explore the appraisal of the COVID-19 infodemic by Romanian healthcare practitioners (HCPs) during 2020, the first year of the pandemic. Since studies on other cultures revealed effects of the infodemic on trust in official policies and health literacy [4,18,22,25,28], we also investigated the satisfaction with governmental measures and the order of vaccinating different population categories. Our results could reveal Romanian HCPs’ perspectives on handling the COVID-19 pandemic, which might show particularities due to this country’s Communist background. Since HCPs are essential agents in the vaccination campaign, our results could have practical implications in assessing the evolution of vaccination awareness in Romania.

### Stress and Mental Health Issues in HCPs Related to COVID-19 Infodemic Exposure

Numerous studies have shown that COVID-19 related fake news on social media or information overload have had a negative impact on the population’s mental health, causing, among others, anxiety or panic [29,30,31,32,33]. Nevertheless, despite the rapidly growing literature on the impact of mass media coverage of the COVID-19 pandemic and mental health, more research into infodemic-related mental health symptoms is needed, especially in populations with direct COVID-19 contact, such as HCPs.

Confronted with the surge of COVID-19 patients and a plethora of professional challenges, HCPs had to manage the growing body of information regarding the virus, treatment methods, and ways to stay safe. Research on various cultures has revealed similarities and differences in HCPs’ approach to the infodemic. For instance, a qualitative study on US participants [34] found that many HCPs appraised information as overflooding, confusing, inaccurate, or biased, despite reporting competence in choosing sources (i.e., high levels of health literacy). A study on SaudiArabian HCPs and non-HCPs revealed a common “neutral” attitude towards false information related to COVID-19 treatment methods, issuing recommendations towards training HCP on filtering information [3]. A study on HCPs in Uganda revealed a high level of agreement with national information guidelines and access to bona fide information despite misinformation endorsed by the general population [35].

In Romania, interesting results published by previous studies encourage further research. For instance, results of a qualitative pilot study on the characteristics of fake online news about COVID-19 [36] revealed a link between the tendency to take health-related decisions based on false online information and lower levels of health literacy in the general population, consistent with other papers [4,22,28]. However, the authors did not investigate the appraisal of information by population categories expected to have higher levels of health literacy, such as HCPs. Another study addressed this issue by investigating differences in stress-related symptoms between HCPs who reported being impacted by fake news on COVID-19 and HCPs who did not [37]. Results indicated that, as the general population got more in touch with misleading health-related information, HCPs were confronted with challenges in their relationships with patients. In addition, higher levels of stress and anxiety were found among HCPs affected by fake news than HCPs who did not report such an impact.

In this framework, more research is needed on the appraisal of mass media information by Romanian HCPs and its impact on their well-being and stress. In addition, while many studies have focused on stress-related symptoms, not many have considered the axiological level (e.g., values) related to COVID-19. Therefore, we formulated the following research objectives:

(1) To explore the appraisal of mass media information by Romanian HCPs and its relation to stress-related variables;
(2) To investigate the position of Romanian HCPs towards governmental measures, vaccination priority, and values.

## 2. Materials and Methods

The present study is part of a project exploring the challenges of HCPs during the COVID-19 pandemic. We designed a cross-sectional survey with 18 items distributed into five sections: (1) demographic data, (2) personal experience with COVID-19, (3) stress and well-being in 2020, (4) ethical principles, and (5) appraisal of mass media information, governmental measures, vaccination priority, and values. The first survey topics were presented in a different article [38]. For the present study, we analyzed results of the fifth section. We investigated mass media information appraisal as predictors of the stress experienced by our respondents.

For the construction of the questionnaire, the authors [38] generated an item pool based on the literature and their practical experience. The research team then evaluated the items for face validity and intelligibility and extracted relevant items for each section. Eight items were chosen to characterize mass-media information and were displayed as multiple-choice formats; items related to stress were presented as Likert scale questions, governmental measures were rated on a 1 to 10 scale, and values and priorities were ranked from 1 to 10.

The targeted population consisted of healthcare professionals who were active throughout 2020, including doctors, nurses, physical therapists, managers, pharmacists, pharmaceutical representatives, psychologists, technicians, and researchers. Respondents were recruited by convenience sampling (i.e., the survey was distributed to members of the authors’ professional networks) and snowball sampling (i.e., participants were asked to further distribute the survey to other HCPs). Despite its shortcomings, this non-probability sampling methodology was considered appropriate for data collection since it facilitated the relatively rapid access to competent HCPs active during 2020 and interested in completing the survey. To avoid a community bias risk, we recruited two initial members from each HCP category who were asked to distribute the survey to other members of their networks. The questionnaire was distributed via social media (i.e., What’s App, Facebook) and email to respondents, who completed it from 03 July 2021 to 05 September 2021. The participants’ anonymity was guaranteed, and no gender and age information were requested. An a priori power analysis using G*Power (Heinrich Heine Universität, Düsseldorf, Germany) revealed that a sample of 122 respondents would be required to achieve a medium effect size and a power of 0.95 for linear regressions [39].

### Data Analysis

We used SPSS for Windows to analyze results (IBM, Armonk, NY, USA). Power analyses were conducted with G*Power. We computed multiple-choice questions as categorical variables and reported frequencies of responses. Central tendencies (i.e., means) and standard deviations were calculated for continuous variables. Linear regression equations were conducted to show relations between variables, and effect sizes (Cohen’s *f*^2^) were computed to show the effect of information appraisal on well-being and stress [40]. Friedman’s ANOVAs were computed to show consistency throughout the sample in ranking values and population categories. Effect size estimates for the Friedman test were calculated as Kendall’s *W* coefficients [41].

## 3. Results

A total of 97 HCPs completed the survey (of which 68% were doctors, 6.2% nurses, 6.2% psychologists and therapists, 5.2% managers, 5.2% workers in the pharmaceutical field, 2.1% academic professionals, and 7.1% had other healthcare professions). The years of experience in the medical field varied from 0–5 (22.7%), 5–10 (11.3%), 10–20 (22.7%), 20–30 (27.8%) to over 30 years of experience (15.5%). Approximately half of the sample (51.5%) worked in inpatient settings, and one quarter (25.8%) in outpatient facilities (see [38]).

### 3.1. Appraisal of Mass Media Information Related to Symptoms of Stress

Frequencies of participants’ responses reflected our sample’s general appraisal of mass media information in 2020, with a considerable proportion attributing negative connotations (see Table 1): mass media information was found exaggerated by half of the sample (52.6%) and confusing by 19.6% of participants. In comparison, only one-quarter of the sample (26.8%) found it useful. Only a small proportion of respondents (5.2%) appraised mass media information as true or helpful for overcoming the pandemic.

Symptoms of stress related to COVID-19 were analyzed and discussed in a different article. Our sample reported a moderate level of well-being and overall moderate levels of irritability, anxiety, and concern, with seldom relaxation difficulties and anticipation of negative events [38].

We computed multiple linear regressions to investigate whether the appraisal of mass media predicted the perceived stress throughout 2020 (see Table 2). No significant regression equations were found for general wellbeing (F(8,88) = 0.613, *p* = 0.764, R^2^ = 0.053), irritability (F(8,88) = 0.902, *p* = 0.185, R^2^ = 0.117), anticipation of a negative event (F(8,88) = 1.379, *p* = 0.217, R^2^ = 0.111), and concern (F(8,88) = 1.167, *p* = 0.079, R^2^ = 0.144). On the other hand, significant regression equations and medium effect sizes were found for relaxation difficulties (F(8,88) = 2.195, *p* = 0.035, R^2^ = 0.166; *f*^2^ = 0.2) and anxiety (F(8,88) = 2.732, *p* = 0.005, R^2^ = 0.213; *f*^2^ = 0.27). At our sample size (*n* = 97), post hoc analyses showed that the achieved power was 0.87 for an effect size of 0.2, and 0.96 for an effect size of 0.27. The negative relations showed that finding mass media information insufficient was associated with less relaxation difficulties, and appraising information as useful was associated with lower levels of anxiety. On the other hand, perceiving information as not good positively predicted relaxation difficulties.

### 3.2. Evaluation of Official Measures, Ranking of Vaccination Priority, and Values

Respondents were asked to appraise the probability of recommending to another state the measures taken by the Romanian government during the pandemic in 2020 on a 1 to 100 scale, 1 indicating the least probability (1%) and 100 indicating the highest probability (100%). The central tendency of the answers was 54.04, with an SD of 27.825, suggesting a moderate level of satisfaction with local measures. A regression equation was performed between the appraisal of mass-media information and the evaluation of governmental measures and showed no significant relations between the variables (F(8,88) = 1.755, *p* = 0.097, R^2^ = 0.371).

Respondents were required to imagine being in a leadership position and rate on a 1 to 100 scale their willingness to sacrifice individual liberty for the common interest in the event of a pandemic. The mean was 71.84, with an SD of 26.578, pointing to our sample’s propensity towards restricting individual rights for the common good.

**Figure 1:**
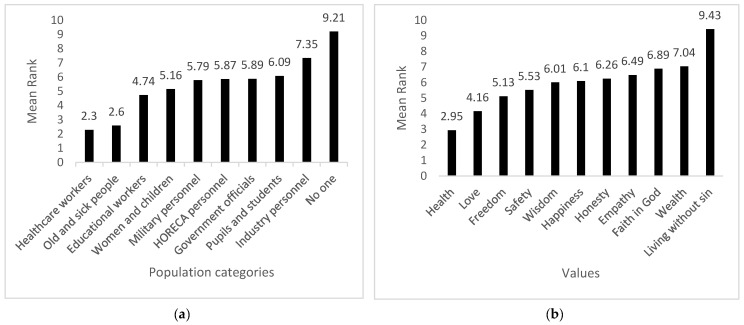
(**a**) Ranking of population categories in the order of vaccination; (**b**) Ranking of values.

Another item requested participants to rank vaccination priority for nine categories of citizens (1 = first to 10 = last). Figure 1a displays the hierarchization of population categories. As expected, healthcare workers were ranked first (mean rank = 2.6), closely followed by old and sick citizens (mean rank = 2.3), with education workers ranked third (mean rank = 4.74). The results of Friedman’s ANOVA revealed significant differences in the ranking of population categories (χ^2^(9) = 394.028, *p* = 0.000), reflecting a moderate agreement among our respondents in attributing vaccination priority (Kendall’s *W* = 0.451). A linear regression equation was computed to verify the relation between vaccination priority and the degree of satisfaction with official measures. No significant relations were found (F(9,87) = 1.743, *p* = 0.091, R^2^ = 0.391).

The final item required participants to rank eleven values according to their importance (1 = most important to 11 = least important). Health was ranked as the most important (mean rank = 2.95), love as the second (mean rank = 4.16), and freedom as the third most important value (mean rank = 5.13). Spiritual and religious values such as faith in God and living without sin were ranked among the least important values (see Figure 1b). Again, the results of Friedman’s ANOVA demonstrated significant differences between values (*χ*^2^(10) = 243.663, *p* = 0.000) and a moderate agreement among our participants in ranking these values (Kendall’s *W* = 0.425). A linear regression equation was computed to show whether the values predicted the tendency of limiting individual freedom, and no significant relations were found (F(10,86) = 1.204, *p* = 0.300, R^2^ = 0.350).

Pearson correlations were computed between the rankings of values and vaccination priority (see Table 3). Significant positive covariations were found for ranking health and vaccinating old and sick people (r = 0.329, *p* < 0.001) among the first options, and for safety and vaccinating industry personnel (r = 0.230, *p* < 0.05). On the other hand, ranking wisdom correlated negatively with vaccinating government officials (r = −0.244, *p* < 0.05). Ranking faith in God among the least important values correlated negatively with vaccinating women and children (r = −0.202, *p* < 0.05). Wealth (r = −0.294, *p* < 0.01) and living without sin (r = −0.203, *p* < 0.01) were ranked as the least important values, which correlated negatively with placing HCPs as the first population category to receive the vaccine.

## 4. Discussion

The present study analyzed results from a cross-sectional survey administered to Romanian healthcare professionals (HCPs) containing items on several issues related to the COVID-19 pandemic in 2020. For the present objectives, we analyzed responses concerning the appraisal of mass media information on COVID-19, the reported symptoms of stress, the evaluation of governmental measures, and the ranking of vaccination priority and moral values.

### 4.1. Evaluation of Mass-MEDIA Information Related to Symptoms of Stress

In line with studies highlighting the overwhelming or inaccurate character of information on public health issues in the COVID-19 era [6,11,12,13,34], overall results in our sample pointed to a primarily negative appraisal of mass-media information. In addition, consistent with previous studies [29,30,32,37], perceiving information as not useful, insufficient, or not good was related to relaxation difficulties and anxiety. Interestingly, a negative relation was found between appraising information as “insufficient” and “useful” and stress-related variables, pointing to a tendency in our sample to experience less stress as the information appeared more useful but also scarcer. Hence, in line with other studies [3,32,33,34], such findings suggest a need for filtered quality information in our HCP sample. In addition, they may indicate a certain detachment from the surge of information characteristic to the COVID-19 pandemic or a need to protect themselves against the infodemic. However, the appraisal of mass media information was not significantly related to the general well-being throughout 2020, which may show effective coping mechanisms or strategies to deal with the infodemic while handling new professional challenges [38].

In addition, unlike other studies reporting relations between trust in official sources and the appraisal of information [16,21,22,25,26], no such relations were found in our sample. Our findings may point to a dissociation between the appraisal of information and governmental measures in our sample of Romanian HCPs.

### 4.2. Evaluation of Official Measures, Vaccination Priority, and Moral Values

Unlike studies indicating high levels of distrust in official or governmental policies in some populations (e.g., in the UK, Turkey, Nigeria) [16,22,25,26], the present results revealed a different perspective of our Romanian HCP sample on the adopted governmental measures. Compared to an overall negative appraisal of mass media information, our sample reported a moderate level of satisfaction with official government actions taken throughout 2020. Such an attitude could have been influenced by the general relaxation of restrictive measures that Romanian HCPs benefited from in 2020 (e.g., mobility allowance). On the other hand, as citizens of a post-Communist country, many with over 20 years of experience (43.3%), our Romanian HCPs might have manifested a traditional position of obedience towards authority, cultivated before the 1989 Revolution [38]. Nevertheless, a study conducted on more than 150 countries since 2020 showed that higher levels of trust within societies led to an increase in resilience and fostered adaptability as the pandemic evolved [42]. Therefore, our sample’s high level of satisfaction with local anti-COVID measures could be interpreted as trust in official measures, contributing to higher adaptability to the epidemiological situation.

The ranking of population categories in the order of vaccination priority revealed our sample’s consistency in choosing healthcare workers as the first option, indicating the prioritization of self-protection in the work field. This finding may point to a self-conserving safety attitude of HCPs, previously found to be negatively linked to symptoms of burnout and stress [38,43]. Next in the hierarchy of vaccination were old and sick people, education workers, and women and children. This ranking was, in fact, consistent with the official Romanian policy on distributing anti-COVID-19 vaccines in 2021: vaccinating healthcare workers in the first phase, followed by citizens of over 60 years of age and patients with comorbidities in the second, and members of the general population in the third phase. However, the lack of significant relations between the appraisal of governmental measures throughout 2020 and the ranking of vaccination priority could suggest that respondents were unaware of this similarity. In addition, at the moment of survey administration, Romania was not dealing with overload in vaccination centers but with general vaccine hesitancy, reaching only 33% of the population vaccinated in mid-September 2021 [44]. Therefore, ranking population categories in the order of vaccination could have been superfluous in a social context marked by resistance to vaccination, possibly as an infodemic effect. Although we did not investigate vaccine acceptance per se, our results contribute to outlining the evolution of vaccine awareness in Romania from an HCP perspective. In other EU countries (e.g., Italy), such investigations have shown progress from low vaccine adherence in 2020 [45] to greater vaccine acceptance in 2021 [18,28], which the authors related to the information strategies within the vaccination campaign [18], or higher educational and health literacy levels [28]. Therefore, in the global epidemiological context, our findings highlight a unique perspective shared by our HCP sample, which indicated satisfaction with local measures and agreement with the Romanian immunization program, despite the negative psychological effects of the infodemic. Given Romania’s current low vaccination incidence [27], future studies on vaccine acceptance could further explore such effects in various Romanian samples as the pandemic progresses. Likewise, the effects of the governmental immunization campaign could be further investigated to keep the public accurately informed and raise vaccine awareness within the population.

Several values showed a common variance with the ranking of vaccinating population categories: ranking health and vaccinating old and sick people among the first options indicated that, in our sample, maintaining health as a primordial value was linked to prioritizing the vaccination of vulnerable categories, such as old and sick people. Safety appeared to share a common variance with vaccinating industry personnel, despite their different hierarchical positions (i.e., safety was ranked fourth and industry personnel last). Two relatively antagonistic values that were ranked last (i.e., wealth and living without sin) correlated negatively with prioritizing HCP vaccination. Such findings indicate that, as the need for self-protection increased, our sample gave less value to wealth and living without sin. Living without sin and faith in God were ranked among the least important values, which may point to a secular status of the medical professions reflected by our sample. However, no consistent relations between values and vaccination categories could be assumed due to the small number of scattered correlations.

From an ethical perspective, the high tendency of our HCP sample to limit individual autonomy for protecting the common interest may signal an authoritarian position. Such a position could be related to assuming the healer role on a personal level, or it could be maintained by a patriarchal medical system that encouraged the patients’ obedience towards the doctor’s authority [38]. These findings indicate a tendency to discount individual liberty in our sample and are in stark contrast to the ranking of values that place freedom third, after health and love. On the other hand, the fact that this tendency was not significantly related to moral values may indicate a dissociation between the personal axiological system and the professional field. Such results are consistent with our previous findings, showing that our HCPs tended to make decisions affecting patients and infringe the bio-ethical principle of autonomy [38]. Therefore, in line with research on other cultures showing various levels of health illiteracy related to COVID-19 [16,22,25,28], our findings could also indicate a certain level of ethical illiteracy in our HCP sample. In this regard, future investigations could focus on comparing HCPs’ levels of ethical literacy across different cultures or comparing perspectives of different professions on ethics and mass media (e.g., HCPs vs. communication specialists) to construct an optimized model of media coverage during the COVID-19 pandemic.

### 4.3. Limitations

Several important limitations are to be mentioned in our study. The first set of limitations is related to our sampling methodology, which restrains result extrapolation to a larger population. The sample size was too small to allow the generalization of results to the entire population of Romanian HCPs. In addition, the fact that most respondents were from the Western part of Romania offers a regional rather than a national perspective on how HCPs appraised the investigated variables. The high proportion of doctors in our sample further restricts result generalizability to other HCP categories. Future studies are encouraged to recruit larger samples of HCPs active nationwide using probability sampling methods (e.g., stratified sampling or systematic sampling), to test our results or compare categories within the healthcare professions.

Second, the cross-sectional design of this study limits the investigation of the appraisal of these variables over time. In this regard, future research could employ longitudinal designs to explore possible shifts in Romanian HCPs’ vision on mass media or vaccination as the COVID-19 pandemic unfolds.

Third, using an online survey to retrospectively investigate the appraisal of mass media, stress, and governmental measures in 2020 might have contributed to increased subjectivity in our sample’s view of last year’s course of events. Other intra-subjective factors like desirability or discounting the importance of questions might have influenced responses. Therefore, future studies could employ psychometric instruments to measure stress and other constructs in Romanian HCPs.

Another limitation of our study is that we did not explore causal relations between variables. Therefore, we cannot infer an influence of mass media information on the mental health status of our respondents. Such relations could be addressed by future studies employing longitudinal or experimental designs to explain human behavior in this period characterized by an abundance of information needing to be filtered.

## 5. Conclusions

In the present study, we analyzed the results of a cross-sectional survey concerning the vision of Romanian HCPs on mass media information related to COVID-19 and the appraisal of governmental measures in 2020. We also investigated the ranking of values and how they related to the hierarchization of population categories in the order of vaccination.

Consistent with previous international and Romanian studies, our sample attributed mostly negative connotations to mass media information on COVID-19 throughout 2020, finding it exaggerated, confusing, false, or not good. While the appraisal of information was not significantly related to the rating of general well-being throughout 2020, it significantly predicted anxiety and relaxation difficulties. Nevertheless, unlike other studies, our HCPs showed a moderate satisfaction with governmental measures during the pandemic in 2020, which in return appeared unrelated to the appraisal of mass media information. Respondents in our sample consistently placed HCPs, old and sick people, and education workers first when evaluating vaccination priority, a ranking showing similarities with the Romanian governmental vaccination program in 2021. Spiritual and religious values were ranked among the last, reflecting a secular status of the healthcare professions. A high tendency to limit individual liberty for the common good appeared in stark contrast with placing freedom third in the hierarchy of values. Such findings indicate an authoritarian position assumed by Romanian HCPs in our sample.

## Figures and Tables

**Table 1:** Frequencies of responses evaluating mass media information.

Mass Media Information Was:	*n*	Percent of Cases
Exaggerated	51	52.6%
Useful	26	26.8%
Confusing	19	19.6%
False	17	17.5%
Not good for me	18	18.6%
Insufficient	10	10.3%
True	5	5.2%
Helpful for overcoming this period	5	5.2%

**Table 2:** Results of multiple regressions—types of information appraisal as predictors of stress-related variables.

Predictors	B	Std. Error	Exp (β)
Relaxation difficulties			
Info useful	−0.259	0.278	−0.133
Info exaggerated	−0.013	0.213	−0.007
Info insufficient	−0.823	0.319	−0.255
Info false	−0.113	280	−0.044
Info true	0.537	0.535	0.121
Info helpful	−0.202	0.445	−0.045
Info not good	0.597	0.269	0.237
Info confusing	0.016	0.257	0.007
Anxiety			
Info useful	−0.748	0.284	−0.322
Info exaggerated	−0.134	0.217	−0.065
Info insufficient	−0.562	0.325	−0.166
Info false	−0.157	0.285	−0.058
Info true	0.497	0.545	0.107
Info helpful	−0.062	0.453	−0.013
Info not good	0.421	0.275	0.159
Info confusing	0.313	0.262	0.121

**Table 3:** Correlations between the ranking of values and vaccination priority.

Values	Vaccination Priority									
	Healthcare Workers	Old and Sick People	Educational Workers	Women and Children	Military Personnel	HORECA Personnel	Government Officials	Pupils and Students	Industry Personnel	No One
Health	0.099	0.329 **	−0.109	0.123	0.025	−0.014	−0.048	−0.039	−0.230 *	−0.173
Love	−0.027	0.016	−0.051	0.103	−0.008	−0.048	−0.002	−0.025	0.051	−0.030
Freedom	0.002	−0.118	−0.148	−0.017	−0.065	−0.064	0.132	0.116	0.062	0.057
Safety	0.147	−0.084	0.155	−0.150	−0.114	−0.047	−0.108	0.002	0.230 *	0.074
Wisdom	0.150	−0.031	0.171	0.085	0.023	−0.022	−0.244 *	−0.028	0.193	−0.188
Happiness	0.049	0.171	−0.009	0.148	−0.169	0.125	−0.079	−0.039	−0.153	−0.067
Honesty	0.013	−0.119	0.156	−0.099	0.189	−0.167	0.063	0.091	0.005	−0.110
Empathy	0.001	0.190	−0.087	0.108	−0.190	0.182	−0.111	0.093	−0.170	−0.033
Faith in God	0.031	−0.117	0.087	−0.202 *	0.148	−0.046	0.079	−0.099	0.019	0.125
Wealth	−0.294 **	0.005	−0.194	0.098	0.085	0.040	0.124	−0.177	0.006	0.206 *
Living without sin	−0.203 *	−0.188	−0.017	−0.097	0.036	0.072	0.171	0.134	−0.030	0.081

** Correlation is significant at *p* < 0.01. * Correlation is significant at *p* < 0.05.

## Data Availability

Data are available upon request.
